# Secondary Subcutaneous *Rhizopus* Infection in a Posttransplant Recipient with Calcific Uremic Arteriolopathy

**DOI:** 10.1155/2020/8695204

**Published:** 2020-06-30

**Authors:** Urmila Anandh, Rakesh Kumar, Vishnu Rao

**Affiliations:** ^1^Dept of Nephrology, Yashoda Hospitals, Alexander Road, Secunderabad 500003, India; ^2^Dept of Infectious Diseases, Apollo Hospitals, Jubilee Hills, Hyderabad 500033, India

## Abstract

Calcific uremic arteriolopathy is a rare condition affecting chronic kidney disease (CKD) patients on long-term dialysis. The clinical manifestations include subcutaneous skin necrosis and ulcers secondary to calcification of the subcutaneous blood vessels. The necrotic tissue often becomes a nidus of infection. The prognosis is often poor. We present a case of a renal allograft recipient who developed a subcutaneous necrotic lesion which was subsequently infected by *Rhizopus* spp. The patient underwent surgical debridement and antifungal therapy. The infection resolved completely. Our case represents agrave underlying condition predisposing a rare and serious posttransplant infection. The outcome was favourable because of early identification and treatment of the infection.

## 1. Introduction

Multiple factors (immunosuppressive medications, renal dysfunction, uncontrolled hyperglycemia, presence of urinary catheters, subcutaneous drains, etc.) are responsible for an increased risk of infection in kidney transplant recipients. Long-term hemodialysis and presence of malnutrition before transplant also have an impact on posttransplant infections [1]. Calcific uremic arteriolopathy (CUA), a complication of long-term hemodialysis, can increase the risk of secondary infections in areas of subcutaneous ischemia and ulcers. Usually, bacterial infections predominate in the early posttransplant period and fungal infections are rare [2]. We present a case of subcutaneous fungal infection in a renal allograft recipient who had calcific uremic arteriolopathy. Despite the gravity of the clinical situation, the outcome was favourable with treatment with antifungals.

## 2. Case Report

**Figure 1:**
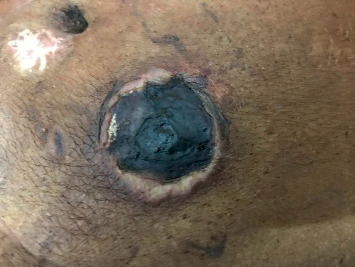
Necrotic nodular lesion on the anterior abdominal wall.

**Figure 2:**
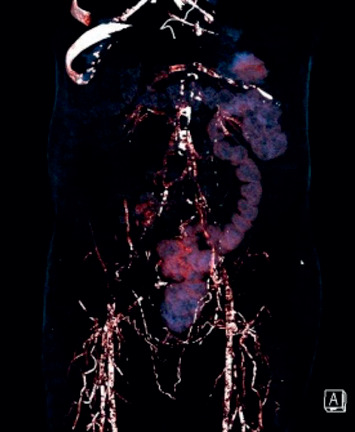
Volume-rendering technique image of the abdominal CT scan showing extensive vascular calcification.

**Figure 3:**
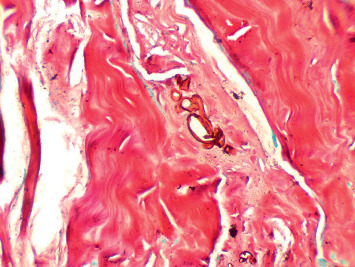
Silver stain of the skin biopsy showing subcutaneous mucormycosis.

**Figure 4:**
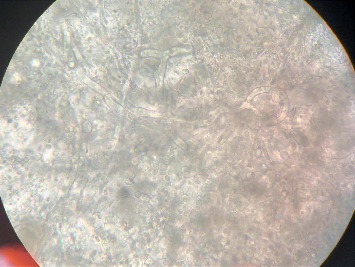
Fungal culture showing broad aseptate hyphae with sporangiophores arising from nodes above the rhizoids suggestive of *Rhizopus* spp.

A 57-year-old male with type 2 diabetes mellitus, coronary artery disease (status postcoronary artery bypass grafting), and chronic kidney disease on dialysis for three years underwent a deceased donor transplantation. His immediate posttransplant period was uneventful, and at discharge, he had normal renal functions. He returned to hospital after 2 weeks with high-grade fever, chills, and allograft dysfunction. He was diagnosed to have allograft pyelonephritis. He was treated with 14 days of intravenous colistin with resolution of his clinical symptoms and allograft dysfunction. His second admission 10 days later was with back pain, and on evaluation, there was evidence of lumbar discitis in the MRI of the lumbar spine. A biopsy of the infected disc was noncontributory, and he was empirically started on ATT and carbapenems based on an infectious disease consult. During his follow-up, he complained of the presence of a new-onset subcutaneous maculonodular necrotic lesion on his abdominal wall (Figure 1). At this time, he had symptoms of abdominal discomfort after eating food. He had evidence of mild allograft dysfunction (creatinine: 2.2 mg/dl), low serum albumin (3.2 g/dl), normal calcium levels (8.10 mg/dl), slightly high phosphorus (5.6 mg/dl), high PTH (572 pg/ml), and normal vitamin D levels (33 ng/ml). His PTH level before transplant was 1029 pg/ml. An allograft biopsy was essentially normal with evidence of resolving pyelonephritis. The abdominal CT scan was noncontributory except for extensive calcification of the intra-abdominal vessels. The anterior abdominal wall vessels were also calcified (Figure 2). A diagnosis of calcific uremic arteriolopathy-associated abdominal wall (subcutaneous) necrosis was made. As the lesion was warm and tender, a diagnosis of secondary infection was considered and a biopsy was taken from the edge of the nodule. The biopsy showed extensive tissue necrosis and presence of nonseptate fungi (Figure 3). The culture isolate was identified as *Rhizopus* spp. (Figure 4). He underwent surgical debridement of the lesion and was treated with 6 weeks of liposomal amphotericin. On completion of therapy, his skin lesion had healed and allograft function was stable (serum creatinine: 1.5 mg/dl).

## 3. Discussion

Fungal infection in the posttransplant period is a rare entity. The cumulative incidence in the first year is 3% [3], with a lower risk for kidney transplants (1.3%) than in other solid organ transplants [4]. The common fungal infections are *Candida*, *Aspergillus*, and cryptococcosis. They occur much later after transplant than bacterial infections [3]. The fungal infections in the tropics have a different epidemiology. Fungi of the Mucorales spp. are important pathogenic organisms besides *Aspergillus*, *Candida*, and *Cryptococcus* [5]. Mucormycosis infection after transplant is often invasive and has a high mortality rate. The most frequent site of involvement is rhinocerebral, followed by pulmonary and cutaneous infections [6].

The risk factors for the development of fungal infections are often a complex interplay of host and environmental factors. The net state of immunosuppression (old age, induction therapy, antirejection therapy, diabetes mellitus, prior antibiotic use, etc.) plays an important factor in the development of these infections [7]. Our patient had almost all predisposing factors which put him at a high risk of developing fungal infections. Presence of necrotic tissue is another predisposing factor for the development of mucormycosis [8]. Our patient had subcutaneous tissue necrosis which was responsible for the secondary *Rhizopus* infection. The subcutaneous necrosis was attributed to his extensive calcific uremic arteriolopathy.

As the infection was localised to an area of necrosis on the anterior abdominal wall and was identified and treated swiftly, the patient had a better outcome than what is observed in renal allograft recipients with mucormycosis [9, 10]. Also, after transplant, the patient's renal function improved which had an ameliorating effect on the underlying calcific uremic arteriolopathy.

## 4. Conclusion

Because of aggressive immunosuppression and use of broad spectrum antibiotics, fungal infections are becoming common in kidney transplant recipients. Early identification of the infection leads to early institution of appropriate therapy. Besides antifungals, surgical debridement is required for the complete resolution of infection as was noted in our case.

## Data Availability

Data are not available in public archives.
